# An unusual postural headache: a case report

**DOI:** 10.1186/s12998-020-00347-0

**Published:** 2020-11-13

**Authors:** Henry Pollard, Rachel Pollard

**Affiliations:** 1 School of Medical and Applied Sciences, CQUniversity Sydney, Level 11, 400 Kent St, Sydney, NSW 2000 Australia; 2 School of Psychology, Faculty of Society & Design, Bond University, 14 University Drive, Robina, QLD 4226 Australia

**Keywords:** Spontaneous intracranial hypotension (SIH), Chiropractic, Postural headache, Telemedicine, Diagnosis, Adverse event

## Abstract

**Abstract:**

**Background:**

This paper presents a case of an evolving unusual thunderclap headache that presented to a chiropractor.

**Case presentation:**

The intense “migraine-like” headache was aggravated by standing up and relieved substantially when lying down. This low pressure, orthostatic headache was diagnosed as a spontaneous intracranial hypotension (SIH) secondary to a spontaneous tear of the dura. It was referred to the local hospital for management with autogolous blood injection to form an epidural blood patch of the defect. It resolved substantially within 3 days.

**Conclusions:**

The significance of key features in the history and examination and how if not recognised and subsequently treated with manual therapy, the dural tear could be attributed to the treatment of the chiropractor, a treatment that would typically involve cervical manipulation. Discussion is provided of the implications of a missed diagnosis and possible subsequent chiropractic management with the evolving SIH being attributed to the chiropractic intervention rather than its true “spontaneous” nature.

## Background

Intense headaches are relatively uncommon and potentially dangerous diagnostic challenges. Whilst there are commonly occurring intense headaches such as migraine, it is the unusual intense headache that presents the diagnostic challenge. It is taught that the practitioner should consider the patient who describes their headache as being “the worst headache that I have ever had” as a cause for concern [1]. Another equally important patient observation is the regular headache sufferer who presents with a “new” headache unlike any before it, especially if that headache is intense or the worse suffered [2].

Determining the nature of the headache is important [3]. Consideration of the location (unilateral, bilateral, focal) the time to onset, the associated symptoms, the association with neck and body (cevicogenic, meningitis, encephalitis) and precipitating red flag conditions are all important.

Red flags in the headache history are of concern and should be screened. Red flags include: (1) systemic symptoms including fever; (2) neoplasm history; (3) neurologic deficit (including decreased consciousness); (4) sudden or abrupt onset; (5) older age (onset after 65 years); (6) pattern change or recent onset of new headache; (7) positional headache; (8) precipitated by sneezing, coughing, or exercise; (9) papilledema; (10) progressive headache and atypical presentations; (11) pregnancy or puerperium; (12) painful eye with autonomic features; (13) posttraumatic onset of headache; (14) pathology of the immune system such as HIV; (15) painkiller overuse or new drug at onset of headache [2].

This paper presents an example of a headache that was both new and intense. Importantly, the headache presented with a key additional feature, that it was aggravated and relieved by a change in posture. Specifically, this case highlights an example of Spontaneous Intracranial Hypotension (SIH) causing an intense headache in a young active male of tall stature who presented remotely to a chiropractor and was referred to the emergency room initially for management with caffeine and later with autologous blood injection into the epidural space.

This case study involves a postural headache caused by a spontaneous leakage of cerebrospinal fluid (CSF). The case highlights the importance of taking a thorough patient history in order to arrive at an accurate diagnosis. Proper diagnosis in this case directed the patient to the correct medical treatment and avoided unnecessary chiropractic treatment.

A history of an intense headache in a patient may have several potential mechanisms and diagnoses. This is true especially for the headache as being described and the worst ever experienced by a patient [3]. The differential diagnosis of the SIH is extensive and is listed in Table 1 from Schievink [4].

**Table 1:**
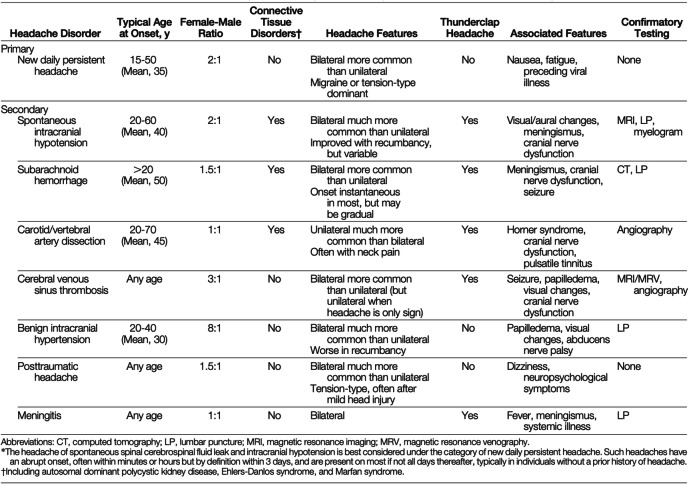
Differential diagnosis of Spontaneous Intracranial Hypotension (SIH) from Schievink [4]

Ref: Schievink WI. Spontaneous spinal cerebrospinal fluid leaks and intracranial hypotension. JAMA. 2006;295(19):2286–96

The incidence of this debilitating condition is estimated to be 5 per 100,000 per year and peaks around the fourth or fifth decades [5] and women are typically more afflicted than men [4].

Connective tissue disorders can be a precipitating factors to SIH [6] and these should be considered in the history.

An important consideration in the development of these headaches are the signs and symptoms associated with the headache (see Table 2) [7]. Key questions to pursue during the clinical encounter include: asking as to the cause of the headache, whether the headache is associated with movement of the head or body (mass effect, space occupying lesion or meningitis/encephalitis) and what aggravates and relieves the headache. In this case, standing upright posture quickly and greatly aggravated the headache (11 out of a score of 10 on a numeric pain rating scale) whilst recumbency significantly (but not totally) relieved it within minutes (1–2 out of a score of 10). Such a headache is typically referred to as a “postural headache” [4].

**Table 2:** Diagnostic criteria for headache due to spontaneous intracranial hypotension [7]

The diagnostic criteria consist of	
A, Orthostatic headache;	
B, The presence of at least one of the following:	
i. low opening pressure (≤ 60 mm H(2) O),	
ii. sustained improvement of symptoms after epidural blood patching,	
iii. Demonstration of an active spinal cerebrospinal fluid leak,	
iv. cranial magnetic resonance imaging changes of intracranial hypotension	
(including brain sagging or pachymeningeal enhancement);	
C, No recent history of dural puncture,	
D, Not attributable to another disorder.	

The taking of a detailed history is essential to determine the diagnosis of SIH [3]. Failure to obtain an appropriate history prior to providing manipulative or other therapy may render the cause of the headache to be attributed to the intervention as has been noted many times in the literature [8–18], even if the intervention was not the cause. Accurate diagnosis after an appropriate history and prior to the provision of manipulative therapy is essential, as noted in the diagnosis described by the paper of Knutson [19] and later discussed by Tuchin [20].

## Case presentation

### History

Patient was a 24-year-old male engineer who presented remotely via telephone (patient was traveling at the time) to his chiropractor with chief complaint of intense headache. This condition began insidiously 24 h earlier. One week prior to the onset of the headache, the patient played semi-elite basketball but denied a traumatic incident. Five days prior to the onset, the patient drove for 12 h and participated in repetitive bending and heavy lifting of furniture.

Pain was located across the parietal and frontal regions of the head. Patient described the pain as intense in nature and rated his symptoms as 11 out of 10 on a numeric pain rating scale. There was associated nausea and vomiting. Patient described the vomiting as nonprojectile. He also was experiencing diaphoresis and a sensation of clamminess across his face. Patient denied dysphagia, dysarthria, dizziness, diplopia or gait irregularities. There was no facial weakness or numbness. There were no prodromal symptoms associated with the headache. Patient denied upper or lower extremity pain, paresthesias or weakness. Cognition, mood and mental functions were normal. Headache pain, nausea and vomiting were aggravated when the patient was upright. Bending over and lying down almost instantaneously relieved the head pain to level 1/10.

### Past medical history

Right olecranon fracture and repair 2017. Appendectomy 4 weeks prior to the onset of his headache. The rest of the health history was normal. Patient took no medications.

### Family history

Patient’s father was 6 ft 9 in. (205.7 cm) without a history of connective tissue disorder.

### Examination (over the phone)

Self-reported height 6.8 in. (205 cm). Patient reported full cervical range of motion which did not aggravate the headache. Bending forward motion with lumbosacral flexion range of motion decreased the headache pain to level 1/10.

### Assessment

Postural headache.

### Plan

Chiropractic physician referred the patient to a nearby hospital for management of a postural headache.

### Progress

Patient reported to a local hospital the following day. He was diagnosed with a headache and was given intravenous fluids and discharged with opioid medication. He initially improved, however, within 2 h the pain worsened again. He telephoned his chiropractor who referred him back to the emergency room with a diagnosis of a postural headache likely due to a CSF leak. Patient decided to consult with a local general practitioner rather than returning to the emergency department. Family physician diagnosed atypical migraine and ordered CT scan of the brain. Patient was seen 2 days later. CT scan of the brain was read as normal. The general practitioner, however, suspected a CSF leak and had the patient transported by ambulance to local hospital emergency department where he was evaluated by a neurologist. During this time, patient’s status deteriorated with persistent intense headaches and multiple episodes of projectile vomiting. MRI of the brain was performed and revealed features of intracranial hypotension with mild diffuse thickening of the pachymeningeal enhancement overlying the brain parenchyma. Neurologist diagnosed spontaneous intracranial hypotension (SIH). Patient was initially placed on caffeine. Following the neurologist diagnosis of SIH, a lumbar puncture was performed (9 days after the onset of the headache). 20 mL of autologous blood was retrieved from the cubital vein and inserted into the epidural space which reproduced his intense headache. He was observed for 6 h following the lumbar puncture and was discharged after headache was reduced to pain level 2–3/10 in the standing position. The headache completely resolved within the following 2 days. Patient remained inactive for 1 week and then resumed short walks. He was advised to avoid lifting or strenuous activity for 1 month. He returned to competitive basketball after 1 month.

## Discussion and conclusions

This case of SIH describes a man of tall stature (204 cm, 6′8″) who presented with an unusual postural headache. Due to the stature of the patient at 6′8″ (204 cm), Beighton’s joint hypermobility score was obtained one month after the recovery when returning to the chiropractor and a score of 2/9 was noted (0–3 normal, 4–9 as representing ligamentous laxity) potentially indicating connective tissue disorders. However, the medical and family history was negative for any connective tissue disorders [21].

A new headache that was described as the worst headache of his life. There was no specific cause despite a recent history of playing a collision sport, repetitive heavy lifting, and a 12 h stressful car drive. He also had a prior history of appendectomy one month before the onset of the headache. Notably, the headache was worsened by standing and relieved by lying down.

This case highlights the value of performing a thorough clinical history. Firstly, to recognise the appropriate symptoms to rule out other serious conditions such as meningitis and secondly, to note the postural nature of the headache.

This case was relatively uncomplicated. However, SIH can present with a range of symptoms and it presents with some or all of the following in up to 50% of cases: hypoacusis, neck stiffness, photophobia and nausea [22].

The mechanism of the symptoms is due to a loss of CSF due to a spontaneous tear in the dura mata. It is possibly the history of heavy lifting and a long stressful drive that may have been the antecedent factor(s) in the onset of the tear (location not identified by MRI) which caused a loss of buoyancy of the brain with a downward pressure of the brainstem through the Foramen Magnum [23].

SIH typically manifests as cerebrospinal fluid (CSF) hypotension and the diagnostic feature includes a postural headache [24]. The etiology of the CSF leakage is not understood completely. Various causes have been attributed to it including: Pre-existing weakness of the dural sac and meningeal diverticula, amongst other causes [25].

The most common associated symptoms in cases of SIH are cranial nerve VI and VIII lesions [4]. Unlike many of the SIH cases that can be found within the literature, there were no cranial nerve symptoms [26]. Nor were there any other cranial nerve symptoms or delirium, mood or loss of consciousness present in this case [4]. This case presented with a relatively slow building headache (of several days duration). However, headaches of this nature can range from mild to incapacitating with symptoms that include coma with a fast onset [27].

The gold standard assessment to aid diagnosis is typically formed from requesting MRI with contrast to locate any possible dural deficits or to visualize the effects of CSF leaks [4].

Management of SIH typically starts with caffeine for reduction of symptoms [4]. Treatment progresses to autogolous injection of 15 to 20 mL of blood into the epidural space in order to promote repair of the dura deficit by coagulants in the blood [28]. This approach is successful in 90% of cases [25, 29], as it was in this case.

Should the autogolous blood fail to improve the condition a larger injection of 20-100 mL can be attempted after a 5 day period [4]. However, if the larger autogolous injection fails, surgery is then performed to repair the defect [30, 31]. This approach is usually successful however in cases where it is not, the prognosis is poor [32].

Most chiropractors provide multimodal treatment of musculoskeletal conditions that includes, manipulation of the spine and extremities, various soft tissue therapies as well as exercise prescription amongst other lifestyle advice [33–35]. However, the profession of chiropractic is frequently referred to as a monotherapy [36] particularly by medicine and other allied health professions [37, 38]. This has resulted in a perception that most if not all chiropractic treatment involves manipulation of the spine [sometimes referred to as an adjustment) [39]. This belief is often further complicated by referring to all failed spinal manipulation (regardless of who performs it) as “chiropractic manipulation”, despite it being referred to just “manipulation” when it is successful [40–44].

Several studies have noted the presence of SIH after spinal manipulative therapy by chiropractors [26, 45] and other practitioners [45]. Some studies have even gone so far as to list “chiropractic neck manipulation” as a risk factor for SIH [46]. We feel this to be inappropriate based on limited research that has focussed on association and not causation.

In summary, this case presents evidence of an evolving migraine like headache that could mistakenly be considered within the scope of practice of chiropractic practice if the postural nature of the headache is not considered. This is due to the migraine-like symptoms presenting with the case and the expectation that management of migraine falls within the scope of chiropractic spinal manipulative therapy [47, 48]. However, we speculate that had the case received chiropractic treatment on the first presentation to the chiropractor with the symptoms that were described as “migraine like” and the condition (SIH) continued to evolve as it did, the following presentation to the emergency room (ER) may have resulted in a subsequent diagnosis of chiropractor induced intracranial headache as previously described in the literature. This case highlights the true diagnosis of a spontaneous condition that had nothing to do with a chiropractic manipulation.

## Data Availability

Not Applicable.
